# Venetoclax plus azacitidine compared with intensive chemotherapy as induction for patients with acute myeloid leukemia: retrospective analysis of an electronic medical record database in the United States

**DOI:** 10.1007/s00277-023-05109-5

**Published:** 2023-02-03

**Authors:** Amer M. Zeidan, Daniel A. Pollyea, Uma Borate, Alberto Vasconcelos, Ravi Potluri, David Rotter, Zephirin Kiendrebeogo, Lona Gaugler, Thomas Prebet, Maria Strocchia, Gaetano Bonifacio, Clara Chen

**Affiliations:** 1 Yale University School of Medicine, New Haven, CT USA; 2 Division of Hematology, School of Medicine, University of Colorado, Aurora, CO USA; 3 Oregon Health & Science University, Portland, OR USA; 4 Bristol Myers Squibb, Princeton, NJ USA; 5 SmartAnalyst Inc., New York, NY USA

**Keywords:** Acute myeloid leukemia, Venetoclax plus azacitidine, Intensive chemotherapy, Clinical outcomes

## Abstract

Intensive chemotherapy (IC) is commonly used to achieve remission in patients with acute myeloid leukemia (AML). Venetoclax plus azacitidine (VEN-AZA) is FDA-approved to treat patients with AML aged ≥ 75 years or who are ineligible for IC. This retrospective analysis used de-identified electronic health records from the US-based Flatiron Health database from patients diagnosed 11/21/2018 to 10/31/2021 to compare treatment outcomes with VEN-AZA vs. IC. Patients were 1:1 propensity score-matched ($N=276$). Assessments included rates of complete remission (CR) and hematopoietic stem cell transplant (HSCT), overall survival (OS), and relapse-free survival (RFS). CR and HSCT rates were higher with IC than with VEN-AZA (60.9% vs. 44.2% [*P* = 0.006] and 18.1% vs. 8.0% [*P* = 0.012], respectively). Median OS was 17.7 months in patients treated with IC and 11.3 months with VEN-AZA without censoring (*P* = 0.278) and 13.7 vs. 10.6 months, respectively, with censoring at HSCT (*P* = 0.584). Median RFS was 12.0 months in patients treated with IC vs. 9.5 months with VEN-AZA without censoring (*P* = 0.431) and 6.4 vs. 7.4 months, respectively, with censoring at HSCT (*P* = 0.444). No OS or RFS differences observed between the two arms reached statistical significance. Randomized controlled trials comparing the two approaches are warranted, as are novel approaches to reduce relapse rates following CR.

**Supplementary Information:**

The online version contains supplementary material available at 10.1007/s00277-023-05109-5.

## Introduction

Many patients with newly diagnosed acute myeloid leukemia (ND-AML) achieve remission with intensive chemotherapy (IC) regimens, followed by hematopoietic stem cell transplant (HSCT); however, the relapse rate is high [1]. Venetoclax (VEN), a *BCL2* inhibitor, in combination with azacitidine (AZA), a hypomethylating agent, is approved to treat older/unfit patients with ND-AML [2–4]. In the landmark VIALE-A trial, combination VEN plus AZA (VEN-AZA) was superior to AZA alone with respect to response rates and overall survival (OS) [4]. Despite the approval of VEN-AZA for a population that is “unfit” for IC, many older adult patients, or those with adverse biology, may be reasonable candidates for either treatment alone [5]. Therefore, it is important to understand the VEN-AZA patterns of use relative to IC in adult patients with ND-AML treated in clinical practice. The objective of this analysis was to describe the clinical outcomes achieved with IC (induction with or without consolidation) or VEN-AZA as first-line therapy for ND-AML.

## Methods

### Data source and study design

This retrospective study used patient data from the Nationwide Flatiron Health electronic medical record (EHR)-derived de-identified database, which sources data from > 2500 oncologists/hematologists and > 280 cancer clinics representing ≥ 2.2 million cancer patients treated in > 800 community and academic sites. Data were derived from structured (i.e., captured directly in the EHR) and unstructured (i.e., non-standardized, such as physician’s notes or pathology reports) sources and externally sourced mortality data.

Eligible patients were aged ≥ 18 years, had ND-AML, and received first-line treatment with VEN-AZA or IC between November 21, 2018, and October 31, 2021. First-line therapy was classified as VEN-AZA if it comprised VEN plus AZA only prior to remission and as IC if the treatment was chemotherapy-based (full list in Supplementary Table 1). Patients diagnosed with acute promyelocytic leukemia or those who participated in a clinical trial during the follow-up period were excluded (Supplementary Fig. 1). Data collection began on cycle 1 day 1 in both treatment groups.

Primary outcomes included rates of complete remission (CR; determined as ≤ 5% blasts in the bone marrow (BM), irrespective of blood count recovery, based on available data) and HSCT at any time among patients with a remission, OS (time from first-line treatment to death), relapse-free survival (RFS; time from first-line treatment to relapse or death), and OS and RFS after censoring at HSCT.

### Propensity-score matching

To address potential confounding due to large differences in baseline characteristics, the VEN-AZA and IC cohorts were propensity-score matched in a 1:1 ratio using nearest-neighbor matching with calipers set to 0.005. A multivariate logistic regression model was used to create the covariates to be matched, resulting in balancing the probability of receiving one or the other treatment and thus mimicking the baseline randomization achieved in a randomized controlled trial (RCT). Patients with missing values were imputed using a multiple imputation by chained equations algorithm. Covariates (at date of diagnosis [index]) were age, sex, race/ethnicity, US geographical region, Charlson comorbidity index, Eastern Cooperative Oncology Group performance status, body mass index, cytogenetic risk category (physician-assessed), white blood cell count > 25,000/μL, whether AML was secondary to myelodysplastic/myeloproliferative diseases (yes or no/unknown), whether AML was treatment-related (yes or no), and whether AML was diagnosed as mixed-phenotype acute leukemia (yes or no).

### Statistical analysis

Categorical variables were compared using a chi-square or Fisher’s exact test; continuous values were compared using a t-test or Mann–Whitney U test. Time-to-event data were estimated using Kaplan–Meier methods, with cohorts compared using log-rank tests. Univariate Cox regression analysis was used to investigate possible treatment-associated influences on OS and RFS in patient subgroups.

## Results

In total, 1209 patients met the selection criteria and 276 were propensity-score matched (138 patients per treatment group; Supplementary Fig. 1). Propensity-score matched cohorts were well balanced for all variables analyzed (Supplementary Table 2). Patients treated with VEN-AZA generally received their initial post–first-line BM assessment later than those treated with IC (35% vs. 68% of patients, respectively, had their first BM assessment within 1 month of first-line therapy (Supplementary Fig. 2)).

Reported CR rates were significantly higher with IC (84/138 [60.9%]) than with VEN-AZA (61/138 [44.2%]; *P* = 0.006; Supplementary Fig. 3). Among patients in remission, rates of HSCT were higher with IC (25/138 [18.1%]) than with VEN-AZA (11/138 [8.0%]; *P* = 0.012).

**Fig. 1:**
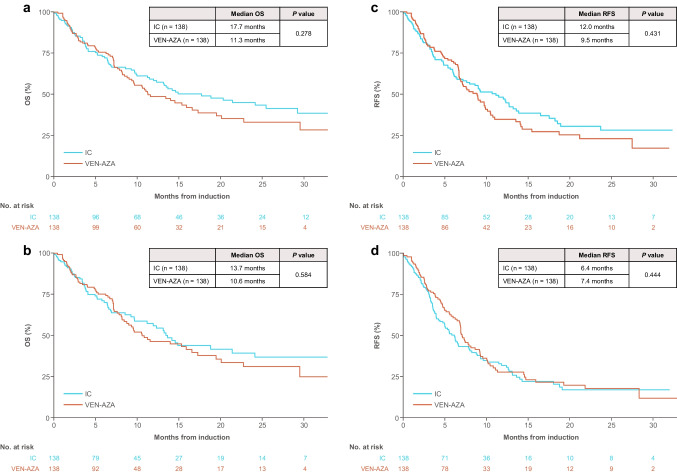
Kaplan–Meier analysis of OS and RFS in propensity-score matched patients with and without censoring at HSCT. **A** OS in all patients. **B** OS in patients after censoring at HSCT^a^. **C** RFS in all patients. **D** RFS in patients after censoring at HSCT^b^. ^a^A total of 30 patients treated with IC and 12 treated with VEN-AZA were censored at HSCT. ^b^For this comparison, 25 patients treated with IC and 12 with VEN-AZA were censored at HSCT. HSCT, hematopoietic stem cell transplant; IC, intensive chemotherapy; OS, overall survival; RFS, relapse-free survival; VEN-AZA, venetoclax plus azacitidine combination therapy

Median OS was 17.7 months with IC and 11.3 months with VEN-AZA overall (*P* = 0.278; Fig. 1A) and 13.7 and 10.6 months, respectively, with censoring at HSCT (*P* = 0.584; Fig. 1B). Median RFS was 12.0 months with IC and 9.5 months with VEN-AZA overall (*P* = 0.431; Fig. 1C) and 6.4 and 7.4 months, respectively, with censoring at HSCT (*P* = 0.444; Fig. 1D). The OS and RFS differences observed between the two arms were not statistically significant.

**Fig. 2:**
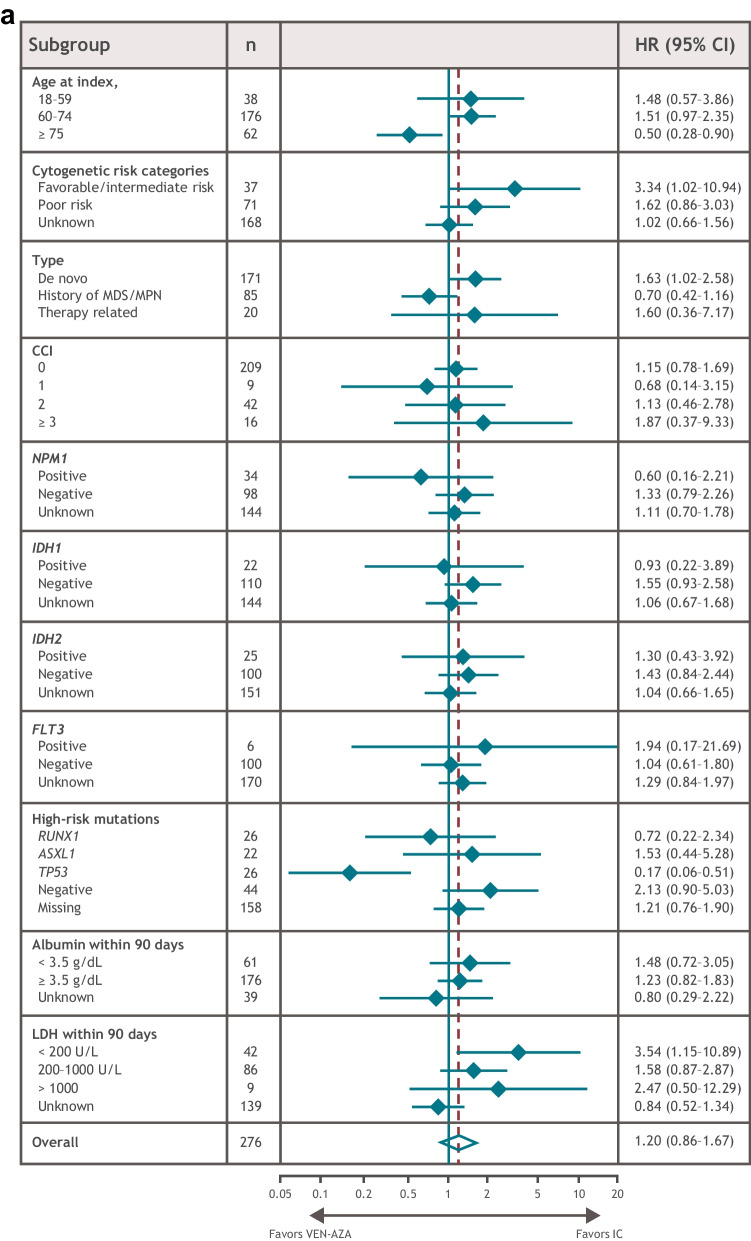

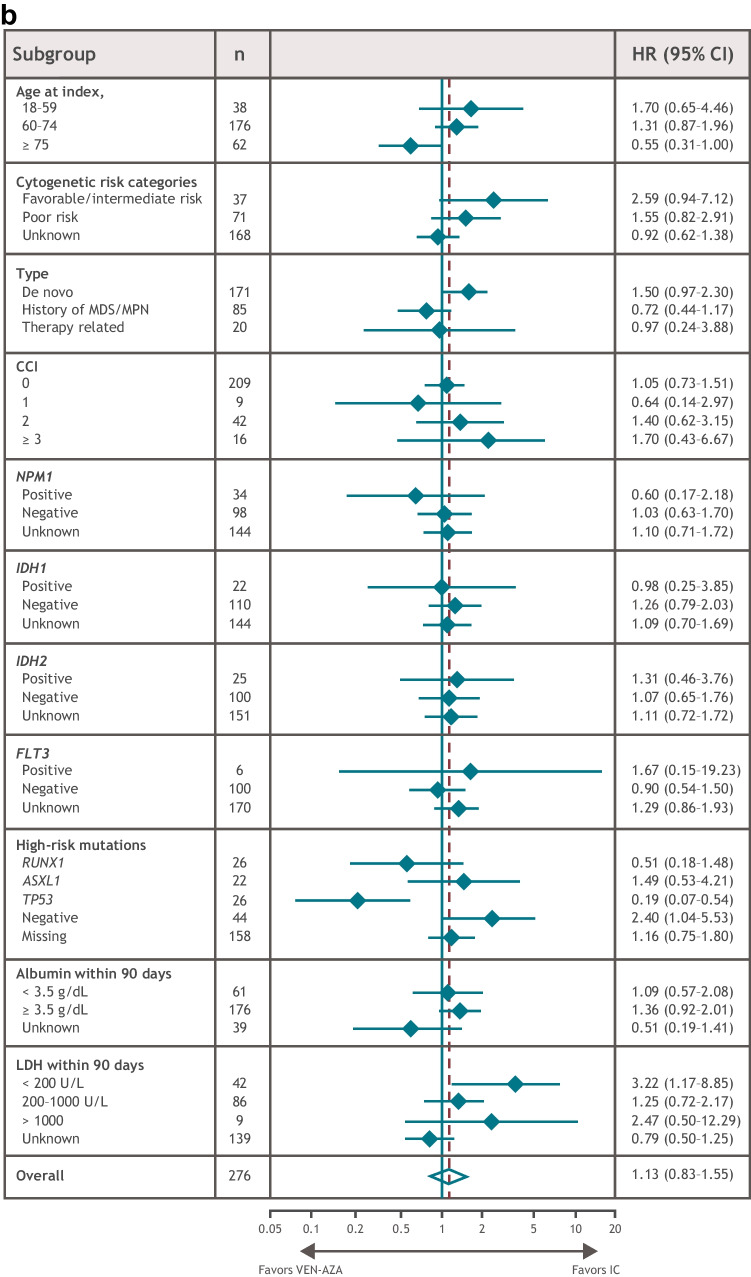
OS and RFS in patient subgroups. **A** OS. **B** RFS. Dashed line represents overall HR. CCI, Charlson comorbidity index; CI, confidence interval; HR, hazard ratio; IC, intensive chemotherapy; LDH, lactate dehydrogenase; MDS/MPD, myelodysplastic/myeloproliferative diseases; OS, overall survival; RFS, relapse-free survival; VEN-AZA, venetoclax and azacitidine combination therapy

In univariate subgroup analyses of the matched patient populations, OS benefit was observed with IC in patients with favorable/intermediate cytogenetic risk, de novo AML, or lactate dehydrogenase (LDH) levels < 200 U/L (Fig. 2A). RFS benefit was observed with IC in patients with no high-risk mutations or with LDH levels < 200 U/L (Fig. 2B). OS and RFS benefit were observed with VEN-AZA in patients with a *TP53* mutation and in those aged ≥ 75 years (Fig. 2A, B). There were no statistically significant differences in high-risk mutation frequency (*RUNX1*, *ASXL1*, or *TP53*) between the matched treatment groups (*P* = 0.232) (Supplementary Table 3), although these data were missing for nearly 60% of patients and were not included in the matching process.

## Discussion

In this real-world analysis of clinical practice treatment for patients with ND-AML, the slight differences between OS or RFS observed between patients treated with VEZ-AZA or IC did not reach statistical significance. The results did indicate that patients treated with IC achieved CR and received subsequent HSCT more frequently than those treated with VEN-AZA. The substantial delay in BM assessment in patients treated with VEN-AZA may have contributed to the lower rates of CR reported with this regimen.

In this analysis, HSCT rates were lower than expected based on clinical standards, particularly in the IC group. This may be due, in part, to the advanced age of the matched population in this cohort (median, 69 years). Because VEN-AZA is approved for patients who are ineligible for IC or aged ≥ 75 years, propensity-score matching may have selected patients in the IC group who were less likely to receive HSCT, possibly due to poor health or comorbidities after induction.

Despite higher initial CR rates, both OS and RFS were not significantly different with IC vs. VEN-AZA, suggesting novel interventions to reduce relapse among patients treated with IC could be an important part of improving OS.

A limitation of this analysis was its observational nature, with increased potential for confounding and treatment selection bias compared with RCTs. Propensity-score matching was performed to offset this bias, but confounding or unknown differences in predictive or prognostic variables may have persisted. Additionally, given that real-world data are collected through EHR extraction, data may be incomplete, and conclusions must be interpreted with caution. Finally, the Flatiron Health EHR-derived de-identified database does not track the number of induction/consolidation cycles patients receive. However, this study was strengthened by including many different treatment regimens used in real-world clinical practice.

## Conclusion

In this retrospective analysis to compare treatment outcomes with VEN-AZA vs. IC, no OS or RFS differences observed between the two arms reached statistical significance. Given the low rate of HSCT observed, further research is necessary to improve survival outcomes. This includes understanding the impact of maintenance therapy such as oral AZA to prolong OS and RFS in patients with AML in remission who do not undergo HSCT [6].

## Supplementary Information

Below is the link to the electronic supplementary material.

Supplementary file1 (DOCX 144 KB)

## Data Availability

The Bristol Myers Squibb policy on data sharing may be found at https://www.bms.com/researchers-and-partners/independent-research/data-sharing-request-process.html.
