# Correction to: Venetoclax plus azacitidine compared with intensive chemotherapy as induction for patients with acute myeloid leukemia: retrospective analysis of an electronic medical record database in the United States

**DOI:** 10.1007/s00277-023-05327-x

**Published:** 2023-06-21

**Authors:** Amer M. Zeidan, Daniel A. Pollyea, Uma Borate, Alberto Vasconcelos, Ravi Potluri, David Rotter, Zephirin Kiendrebeogo, Lona Gaugler, Thomas Prebet, Maria Strocchia, Gaetano Bonifacio, Clara Chen

**Affiliations:** 1 Yale University School of Medicine, New Haven, CT USA; 2 Division of Hematology, School of Medicine, University of Colorado, Aurora, CO USA; 3 Oregon Health & Science University, Portland, OR USA; 4 Bristol Myers Squibb, Princeton, NJ USA; 5 SmartAnalyst Inc., New York, NY USA

**Correction to: Annals of Hematology**

10.1007/s00277-023-05109-5

The article “Venetoclax plus azacitidine compared with intensive chemotherapy as induction for patients with acute myeloid leukemia: retrospective analysis of an electronic medical record database in the United States”, written by Amer M. Zeidan, Daniel A. Pollyea, Uma Borate, Alberto Vasconcelos, Ravi Potluri, David Rotter, Zephirin Kiendrebeogo, Lona Gaugler, Thomas Prebet, Maria Strocchia, Gaetano Bonifacio and Clara Chen, was originally published Online First without Open Access. After publication in volume 102, issue 4, page 749–754 the author decided to opt for Open Choice and to make the article an Open Access publication. Therefore, the copyright of the article has been changed to © The Author(s) 2023 and the article is forthwith distributed under the terms of the Creative Commons Attribution 4.0 International License, which permits use, sharing, adaptation, distribution and reproduction in any medium or format, as long as you give appropriate credit to the original author(s) and the source, provide a link to the Creative Commons licence, and indicate if changes were made. The images or other third party material in this article are included in the article’s Creative Commons licence, unless indicated otherwise in a credit line to the material. If material is not included in the article’s Creative Commons licence and your intended use is not permitted by statutory regulation or exceeds the permitted use, you will need to obtain permission directly from the copyright holder. To view a copy of this licence, visit http://creativecommons.org/licenses/by/4.0.

The original article has been corrected.

