# Supplementary material for: Venetoclax plus azacitidine compared with intensive chemotherapy as induction for patients with acute myeloid leukemia: retrospective analysis of an electronic medical record database in the United States
Source: Ann Hematol. 2023 Feb 3;102(4):749–54. doi: 10.1007/s00277-023-05109-5 (PMC10285011; doi:10.1007/s00277-023-05109-5)
Supplement: Supplementary file 1 — Supplementary file1 (DOCX 144 KB) [file 277_2023_5109_MOESM1_ESM.docx]

**Supplementary Table 1** Induction/consolidation chemotherapy regimens in IC group

| **IC regimen** | **IC (*n* = 138)**  **n (%)** |
| --- | --- |
| Cytarabine, daunorubicin | 22 (15.9) |
| 7+3 not otherwise specified | 15 (10.9) |
| Cytarabine | 12 (8.7) |
| Cytarabine, idarubicin | 12 (8.7) |
| Cytarabine, fludarabine, idarubicin | 8 (5.8) |
| Cytarabine, fludarabine | 5 (3.6) |
| Cytarabine, hydroxyurea | 5 (3.6) |
| Cytarabine, hydroxyurea, idarubicin | 5 (3.6) |
| Cytarabine, daunorubicin, midostaurin | 4 (2.9) |
| Cytarabine, glasdegib | 4 (2.9) |
| Cytarabine, idarubicin, midostaurin | 4 (2.9) |
| 7+3 not otherwise specified, cytarabine, daunorubicin | 3 (2.2) |
| Cytarabine, hydroxyurea, idarubicin, midostaurin | 3 (2.2) |
| 7+3 not otherwise specified, cytarabine, daunorubicin, midostaurin | 2 (1.4) |
| Cytarabine, daunorubicin, daunorubicin/cytarabine liposomal | 2 (1.4) |
| Cytarabine, daunorubicin, gemtuzumab ozogamicin | 2 (1.4) |
| Cytarabine, daunorubicin, hydroxyurea | 2 (1.4) |
| 7+3 not otherwise specified, cladribine, cytarabine, mitoxantrone | 1 (0.7) |
| 7+3 not otherwise specified, cytarabine, fludarabine | 1 (0.7) |
| 7+3 not otherwise specified, cytarabine, fludarabine, hydroxyurea | 1 (0.7) |
| 7+3 not otherwise specified, dasatinib | 1 (0.7) |
| 7+3 not otherwise specified, hydroxyurea, midostaurin | 1 (0.7) |
| 7+3 not otherwise specified, midostaurin | 1 (0.7) |
| Anastrozole, cladribine, cytarabine, hydroxyurea | 1 (0.7) |
| Cladribine, cytarabine | 1 (0.7) |
| Cladribine, cytarabine, idelalisib | 1 (0.7) |
| Cladribine, cytarabine, midostaurin | 1 (0.7) |
| Cladribine, cytarabine, mitoxantrone | 1 (0.7) |
| Cladribine, cytarabine, sorafenib | 1 (0.7) |
| Clofarabine, cytarabine | 1 (0.7) |
| Cyclophosphamide, cytarabine, doxorubicin, mesna, methotrexate, vincristine | 1 (0.7) |
| Cyclophosphamide, cytarabine, idarubicin, mesna, midostaurin | 1 (0.7) |
| Cytarabine, dasatinib, fludarabine | 1 (0.7) |
| Cytarabine, dasatinib, idarubicin, sorafenib | 1 (0.7) |
| Cytarabine, daunorubicin, gemtuzumab ozogamicin, medroxyprogesterone | 1 (0.7) |
| Cytarabine, daunorubicin, hydroxyurea, idarubicin | 1 (0.7) |
| Cytarabine, daunorubicin, hydroxyurea, midostaurin | 1 (0.7) |
| Cytarabine, daunorubicin/cytarabine liposomal | 1 (0.7) |
| Cytarabine, daunorubicin/cytarabine liposomal, fludarabine, idarubicin | 1 (0.7) |
| Cytarabine, daunorubicin/cytarabine liposomal, methotrexate | 1 (0.7) |
| Cytarabine, enasidenib mesylate | 1 (0.7) |
| Cytarabine, fludarabine, gemtuzumab ozogamicin | 1 (0.7) |
| Cytarabine, fludarabine, hydroxyurea, idarubicin | 1 (0.7) |
| Cytarabine, hydroxyurea, idarubicin, methotrexate, mitoxantrone | 1 (0.7) |
| Cytarabine, idarubicin, methotrexate | 1 (0.7) |

7+3, 7 days of cytarabine + 3 days of anthracycline; IC, intensive chemotherapy.

**Supplementary Table 2** Baseline characteristics

|  | **Before matching** | | | **After matching** | | |
| --- | --- | --- | --- | --- | --- | --- |
|  | **VEN-AZA**  **(*n* = 592)** | **IC**  **(*n* = 617)** | ***P*** | **VEN-AZA**  **(*n* = 138)** | **IC**  **(*n* = 138)** | ***P*** |
| Age at index, years  Mean (SD)  Median (IQR) | 75.0 (7.7)  76.0 (9.5) | 55.4 (14.2)  58.0 (19.0) | < 0.001 | 68.7 (10.1)  71.0 (12.0) | 68.6 (8.6)  69.0 (10.0) | 0.447 |
| Sex, n (%)  Female  Male | 226 (38.2)  366 (61.8) | 265 (42.9)  352 (57.1) | 0.091 | 53 (38.4)  85 (61.6) | 60 (43.5)  78 (56.5) | 0.392 |
| Mean (SD) CCI | 0.7 (1.4) | 0.4 (1.0) | 0.052 | 0.5 (1.1) | 0.6 (1.2) | 0.651 |
| Most recent ECOG PS,^a^ n (%)  0  1  ≥ 2  Unknown | 105 (17.7)  221 (37.3)  117 (19.8)  149 (25.2) | 130 (21.1)  162 (26.3)  61 (9.9)  264 (42.8) | < 0.001 | 30 (21.7)  44 (31.9)  18 (13.0)  46 (33.3) | 26 (18.8)  39 (28.3)  22 (15.9)  51 (37.0) | 0.742 |
| Most recent BMI to index date, n (%)  Underweight  Normal weight  Overweight  Obese  Unknown | 11 (1.9)  166 (28.0)  194 (32.8)  177 (29.9)  44 (7.4) | 9 (1.5)  140 (22.7)  174 (28.2)  218 (35.3)  76 (12.3) | 0.003 | 1 (0.7)  36 (26.1)  42 (30.4)  46 (33.3)  13 (9.4) | 2 (1.4)  34 (24.6)  38 (27.5)  53 (38.4)  11 (8.0) | 0.869 |
| AML diagnosis preceded by MDS/MPD  Yes (vs no/unknown), n (%) | 241 (40.7) | 74 (12.0) | < 0.001 | 44 (31.9) | 44 (31.9) | 1.000 |
| Cytogenetic risk category,^b^ n (%)  Favorable/low risk  Intermediate risk  Poor/adverse/high risk  Unknown | 17 (2.9)  36 (6.1)  177 (29.9)  362 (61.1) | 98 (15.9)  82 (13.3)  129 (20.9)  308 (49.9) | < 0.001 | 6 (4.3)  15 (10.9)  30 (21.7)  87 (63.0) | 9 (6.5)  7 (5.1)  41 (29.7)  81 (58.7) | 0.143 |
| AML was treatment related  Yes (vs no), n (%) | 59 (10.0) | 26 (4.2) | < 0.001 | 10 (7.2) | 10 (7.2) | 1.000 |
| WBC count > 25,000/μL, n (%)  No  Yes  Unknown | 378 (63.9)  60 (10.1)  154 (26.0) | 196 (31.8)  92 (14.9)  329 (53.3) | < 0.001 | 66 (47.8)  18 (13.0)  54 (39.1) | 68 (49.3)  22 (15.9)  48 (34.8) | 0.676 |
| Race/ethnicity, n (%)  White  Black  Asian  Other race  Hispanic/Latino  Unknown | 360 (60.8)  34 (5.7)  10 (1.7)  113 (19.1)  18 (3.0)  57 (9.6) | 353 (57.2)  43 (7.0)  15 (2.4)  92 (14.9)  29 (4.7)  85 (13.8) | 0.037 | 83 (60.1)  9 (6.5)  3 (2.2)  16 (11.6)  8 (5.8)  19 (13.8) | 80 (58.0)  11 (8.0)  3 (2.2)  24 (17.4)  5 (3.6)  15 (10.9) | 0.697 |
| AML diagnosed as mixed phenotype  Yes (vs no), n (%) | 6 (1.0) | 14 (2.3) | 0.087 | 2 (1.4) | 1 (0.7) | 0.562 |
| US region, n (%)  Northeast  Midwest  South  West  Puerto Rico  Unknown | 63 (10.6)  52 (8.8)  275 (46.5)  65 (11.0)  5 (0.8)  132 (22.3) | 25 (4.1)  69 (11.2)  252 (40.8)  77 (12.5)  2 (0.3)  192 (31.1) | < 0.001 | 8 (5.8)  13 (9.4)  63 (45.7)  12 (8.7)  2 (1.4)  40 (29.0) | 6 (4.3)  16 (11.6)  58 (42.0)  13 (9.4)  1 (0.7)  44 (31.9) | 0.928 |

^a^Within 90 days of diagnosis. ^b^As documented by a physician. AML, acute myeloid leukemia; BMI, body mass index; CCI, Charlson comorbidity index; ECOG PS, Eastern Cooperative Oncology Group performance status; IC, intensive chemotherapy; IQR, interquartile range; MDS/MPD, myelodysplastic/myeloproliferative diseases; SD, standard deviation; VEN-AZA, venetoclax plus azacitidine combination therapy; WBC, white blood cell.

**Supplementary Table 3** Frequency of high-risk mutations in the analysis cohorts

| **High-risk mutations** | **VEN-AZA**  **(*n* = 138),**  **n (%)** | **IC**  **(*n* = 138),**  **n (%)** | ***P*** |
| --- | --- | --- | --- |
| *RUNX1*  *ASXL1*  *TP53*  Negative  Missing | 10 (7.2)  12 (8.7)  17 (12.3)  18 (13.0)  81 (58.7) | 16 (11.6)  10 (7.2)  9 (6.5)  26 (18.8)  77 (55.8) | 0.232 |

IC, intensive chemotherapy; VEN-AZA, venetoclax plus azacitidine combination therapy.

**Supplementary Fig. 1**  **Patient selection.**


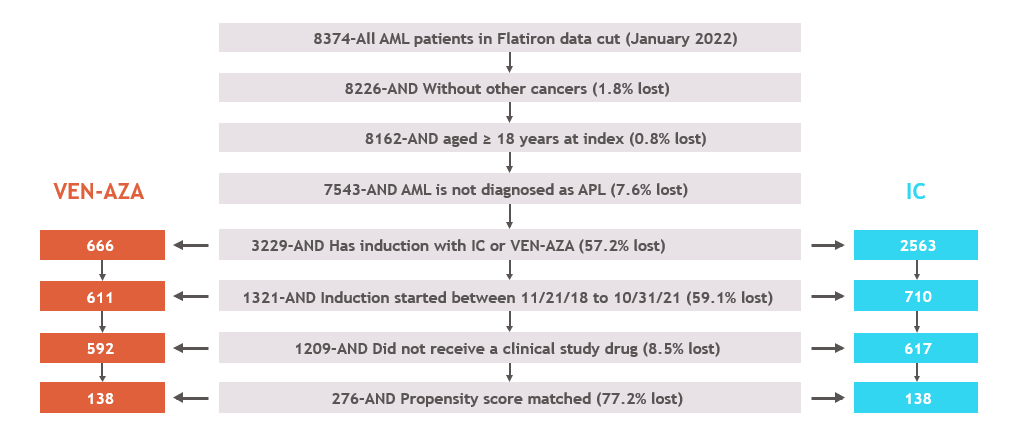


AML, acute myeloid leukemia; APL, acute promyelocytic leukemia; IC, intensive chemotherapy; VEN-AZA, venetoclax plus azacitidine combination therapy.

**Supplementary Fig. 2**  **Rates^a^ of bone marrow assessment post–first-line therapy.**


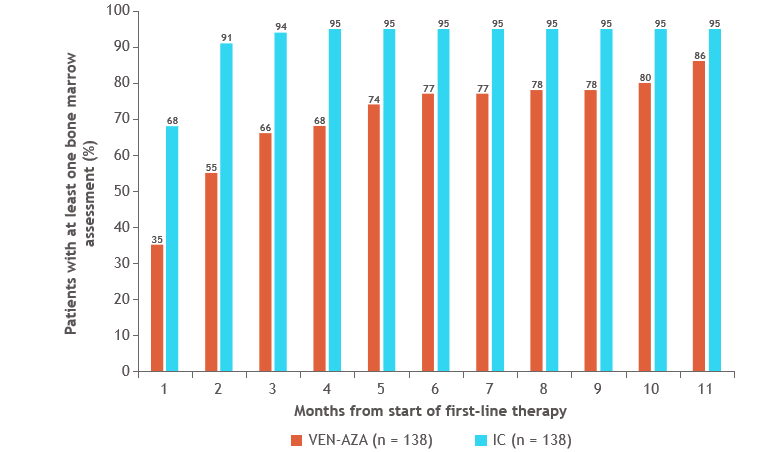


^a^Rates were calculated by dividing the number of patients with at least 1 bone marrow assessment over the number of patients still in follow-up at the end of the calendar month post-initiation of first-line therapy (i.e., *N* = 138 at treatment start); monthly rates were culminated to derive subsequent rates. IC, intensive chemotherapy; VEN-AZA, venetoclax plus azacitidine combination therapy.

**Supplementary Fig. 3 Rates of remission and transplant**


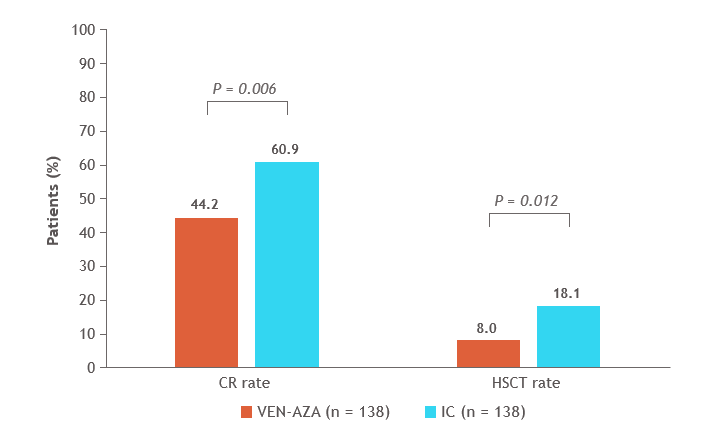


CR, complete remission; IC, intensive chemotherapy; HSCT, hematopoietic stem cell transplant; VEN-AZA, venetoclax plus azacitidine combination therapy.
